# Increased glomerulonephritis recurrence after living related donation

**DOI:** 10.1186/s12882-016-0435-z

**Published:** 2017-01-17

**Authors:** A. L. Kennard, S. H. Jiang, G. D. Walters

**Affiliations:** 1 Department of Renal Medicine, The Canberra Hospital, PO Box 11, Woden, ACT 2605 Australia; 2 Department of Immunology and Infectious Diseases, John Curtin School of Medical Research, Australian National University, Canberra, Australia; 3 Australian National University Medical School, Garran, Australia; 4 ANZDATA Registry, Adelaide 5000, Australia

**Keywords:** Kidney, Donor, Transplant, Survival, Glomerulonephritis, Recurrence

## Abstract

**Background:**

Kidney transplantation confers superior outcomes for patients with end stage kidney disease, and live donor kidneys associate with superior outcomes compared to deceased donor kidneys. Modern immunosuppression has improved rejection rates and transplant survival and, as a result, recurrence of glomerulonephritis has emerged as a major cause of allograft loss. However, many glomerulonephritides have significant genetic risk which may manifest through kidney intrinsic or systemic mechanisms. We hypothesise that heritable kidney intrinsic predisposition to glomerulonephritis will result in increased risk of glomerulonephritis recurrence in kidneys transplanted from genetically related donors.

**Methods:**

We investigated the effect of living related donation on rates of recurrence and subsequent graft outcomes in 7236 patient from 28 years of ANZDATA transplant registry data. Data were analysed in R, using Kaplan Meier Survival analysis and adjusted analyses performed using Cox Proportional Hazards methods. A competing risk model was also analysed.

**Results:**

Glomerulonephritis recurrence rates were significantly higher in living related donor grafts compared to either living unrelated or deceased donor grafts (*p* < 0 · 001). In IgA nephropathy, transplantation from living related donor kidneys demonstrated a 10 year recurrence rate of 16 · 7% compared to 7 · 1% in living unrelated donors and 9 · 2% in deceased donors (HR:1 · 7, 95% CI:1 · 26–2 · 26, *p* = 0 · 0005 for living related vs deceased donors). In focal segmental glomerulosclerosis, risk of recurrence at 10 years was 14 · 6% in living related donors compared to 10 · 8% in living unrelated donors and 6 · 6% in deceased donors (HR:2 · 2, 95% CI 1 · 34–3 · 64, *p* = 0 · 002) for living related vs deceased donors. Primary glomerulonephritis death censored graft survival was superior for living donor grafts, related or unrelated, compared to deceased donor grafts.

**Conclusions:**

We identified a significant increase in the risk of glomerulonephritis recurrence in IgA Nephropathy and Focal Segmental Glomerulosclerosis in living related donors compared to a deceased donors.

## Background

Kidney transplantation confers the best prognosis [1] for patients with end stage renal failure and living kidney donation confers superior outcomes to deceased kidney donation [2]. However, modern immunosuppression has substantially improved rejection rates and as a result GN recurrence is increasingly a major cause of allograft loss [3]. While conflicting reports suggested that recurrence of GN may [4, 5] or may not [6, 7] significantly reduce graft survival with post-transplant GN recurrence the third most common cause of allograft loss [3, 6, 8].

It has been observed that kidneys transplanted from living donors have [9] both earlier onset [10–12] and increased rates [4, 5, 7, 13–17] of GN recurrence. Most glomerulonephritides have been associated with significant genetic risks [9, 10]. It is unclear how this heritability contributes to disease pathogenesis, with both kidney-intrinsic [11] or systemic [12] mechanisms implicated. Equally, it is unclear whether the increased risk of GN recurrence is due to live donation per se or the presence of related donors in the donor population [18–21]. Furthermore, it is unclear what effect increased recurrence has on the superior outcomes typically associated with living related donation. In studies reporting increased risks of GN recurrence with living related donation, living related and unrelated graft survival was similar at 5 and 10 years [14, 22], whereas other studies report preserved survival advantage with living related donation regardless of GN recurrence [18, 23, 24].

We have investigated the effect of living related donation on rates of GN recurrence and subsequent graft outcomes from 28 years of ANZDATA transplant registry data, comparing living related, living unrelated and deceased donors.

## Methods

### Study population

The Australia and New Zealand Dialysis and Transplant Registry (ANZDATA) collects data on all renal transplants performed in Australia and New Zealand. Baseline clinical and demographic data are recorded with follow up data collected every 6 months from all transplant centres. Patient anonymity is assured with coding of data on entry. Recurrence of glomerulonephritis is recorded in the registry as the date of the relevant biopsy. Biopsy practice is defined by each centre according to its current clinical practice. This will necessarily have changed over the last 30 years with some centres moving to protocol biopsies. Biopsy indication is not available for this population currently. Data were extracted from ANZDATA for all renal allografts transplanted for patients with a primary GN between 1985 and 2013 within Australia and New Zealand. Transplants included first, second, or subsequent transplants. Primary end points were death censored graft loss (DCGS) or GN recurrence until December 2013.

### Statistical analyses

All statistical analyses were carried out in R [13]. Baseline characteristics among groups were assessed using Pearson’s chi-square test and one way ANOVA. In analyses of “Primary Glomerulonephritis” we included only patients with IgA Nephropathy (IGAN), Focal Segmental Glomerulosclerosis (FSGS), Membranous Nephropathy (MN) and Mesangiocapillary Glomerulonephritis (MCGN), excluding those coded with “Other GN”. Survival analyses were performed in R, using the Surv() function from the survival library [14] and the npsurv() and survplot() functions from the rms library [15]. Graphs are plotted with 95% confidence intervals. Cox models were constructed to account for confounding due to differences across groups. Models adjusted for age, sex, dialysis vintage, HLA mismatch, peak PRA, total ischemic time and graft number. Cox proportional hazards ratios (HRs) are calculated using the coxph() function from the survival library. Data were analysed using a competing risk model using the mutually exclusive outcomes of Death before Recurrence (DbR), Graft Failure before Recurrence (GFbR) and Recurrence utilising the Surv() function with type=”mstate”.

## Results

### Baseline demographics

Sixteen thousand twenty-three renal transplants were performed between 1985 and 2013 of which 7,236 (45 · 16%) were for biopsy-proven primary glomerulonephritis (GN). Characteristics of the patients are summarised in Table 1. The most common primary GN was IgA Nephropathy (IGAN) (33%) followed by Focal Segmental Glomerulosclerosis (FSGS) (13 · 4%). Amongst patients with primary GN (IGAN, FSGS, Membranous Nephropathy (MN) and Mesangiocapillary (MCGN) only) 2, 693 (66 · 9%) transplants were from deceased donors, 892 (22 · 2%) from living related donors and 440 (10 · 9%) were recipients from living unrelated donors.

**Table 1:** Chracteristics of GN Patients by donor category

	Deceased	Related	Unrelated	Total	*P* value*
GN Patients	4956	1576	704	7236	0
Other Patients	6201	1686	900	8787	
Total	11157	3262	1604	16023	
GN patients					
Age (Mean, SD)	45.2 (13.4)	35.5 (13.7)	49.4 (12)*	43.6 (15.2)	<0.0001
GN Category					
FSGS	671 (13.5)	207 (13.1)	97 (13.7)	975 (13.4)	<0.0001
IgA	1552 (31.3)	557 (35.3)	284 (40.3)	2393 (33)	
MCGN	260 (5.2)	57 (3.6)	31 (4.4)	348 (4.8)	
MN	210 (4.2)	71 (4.5)	28 (3.9)	309 (4.2)	
Other GN	2263 (45.6)	684 (43.4)	264 (37.5)	3211 (44.3)	
Male Gender, n (%)	3311 (66.8)	994 (63)	487 (69.1)	4792 (66.2)	<0.005
Caucasian ethnicity, n (%)	4003 (80.7)	1283 (81.4)	586 (83.2)	5872 (81.1)	0.0006
Graft Number, n (%)					
Primary	4385 (88.4)	1464 (92.8)	626 (88.9)	6475 (89.4)	<0.0001
Secondary	514 (10.3)	100 (6.3)	72 (10.2)	686 (9.4)	
Subsequent	57 (1.1)	12 (0.7)	6 (0.8)	75 (1)	
Diabetes, n (%)	485 (9.7)	62 (3.9)	45 (6.3)	592 (8.1)	<0.0001
Peak panel reactive antibodies (%), Median (IQR)	5 (0–26)	1 (0–10)	0 (0–8)	3 (0–20)	<0.001
Total ischaemic time (hrs), Median (IQR)	14 (11–18)	2 (1–3)	2 (1–4)	11 (3–16)	<0.001
Zero HLA mismatches, n(%)	219 (4.4)	280 (17.7)	35 (4.9)	534 (7.4%)	<0.0001

*p values calculated with Student's t test for continuous variables and Chi squared test for categorical variables

### Increased glomerulonephritis recurrence in allografts from living related donors

**Fig. 1:**
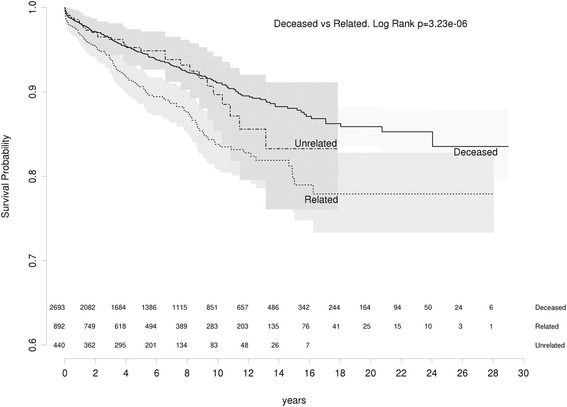
All GNs: recurrence free survival living related vs living unrelated vs deceased

GN recurrence occurred in 424 (10 · 5%) of all primary GN allografts. GN recurrence rates were significantly higher in living related donor grafts compared to either living unrelated or deceased donor grafts (*p* < 0 · 001) (Fig. 1). At 10 years, 16 · 2% of living related grafts had recurrent GN compared to 10 · 3% of living unrelated and 8 · 9% of deceased donor grafts. There was significantly increased risks of GN recurrence with living related donor kidneys compared with deceased donor kidneys (HR:1 · 7, 95% CI:1 · 4–2 · 1, *p* < 0 · 00001) or living unrelated donor kidneys (HR: 1 · 6, 95% CI:1 · 05–2 · 4, *p* = 0 · 03). A competing risk model was used to compare rates of recurrence in deceased and related donor allografts. Ten year recurrence rates were slightly reduced at 7.5% for deceased and 14.8% for related donors but they remained statistically significant. These are compared with standard survival analysis results in Table 2.

**Table 2:** Recurrence rates by standard survival analysis and competing risk analysis. Mutually exclusive risks of Death before Recurrence, Graft Failure before Recurrence and Recurrence were used to construct the competing risk analysis

	Competing risk analysis		Standard survival analysis	
Time (years)	Deceased	Related	Deceased	Related
5	4.8	9	5.3	9.4
10	7.5	14.8	8.9	16.2
15	9.3	17.7	11.9	20.3
20	10.2	18.8	14.1	22.1

**Fig. 2:**
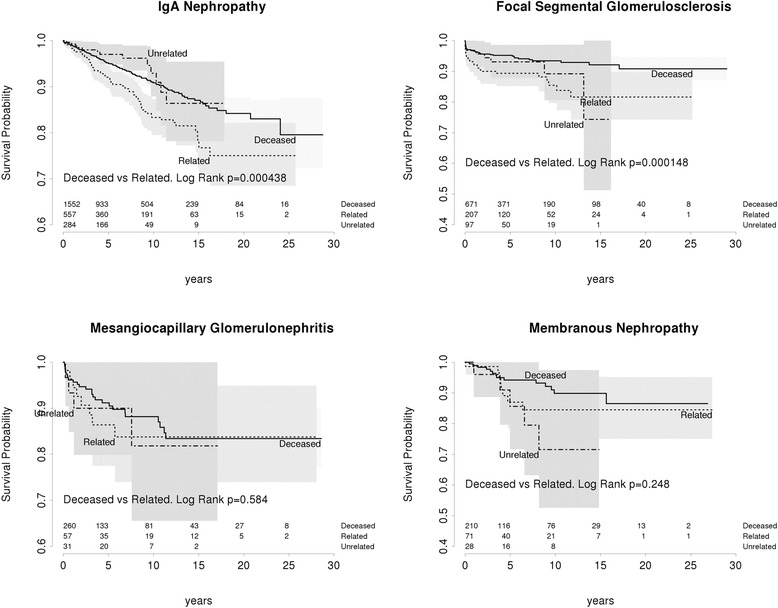
Recurrence free survival in each GN

IGAN and FSGS demonstrated increased rates of recurrence associated with living related donation. For IGAN, living related donor kidneys demonstrated a 10 year recurrence rate of 16 · 7% (recurrence free survival 83 · 3% (95% CI 79 · 5–87 · 3)) compared to 7 · 1% (recurrence free survival 92 · 9% (95% CI 87 · 9–98 · 2)) for living unrelated donors and 9 · 2% (recurrence free survival 90 · 8 (95% CI 89–92 · 7)) for deceased donors. In adjusted analysis, the risk of IGAN recurrence was significantly increased for living related donor kidneys compared to either deceased donor kidneys (HR:1 · 7, 95% CI:1 · 26–2 · 26, *p* = 0 · 0005) or living unrelated donor kidneys (HR:2 · 2, 95% CI:1 · 2–4 · 0, *p* = 0 · 009) (Fig. 2).

In FSGS, living related grafts had an elevated risk of recurrence at 10 years of 14 · 6% (recurrence free survival 85 · 4%, 95% CI 79 · 6–91 · 6) compared to 10 · 8% among living unrelated donors (recurrence free survival 89 · 2%, 95% CI 80 · 6–98 · 7) and 6 · 6% in deceased donors (recurrence free survival 93 · 4% 95% CI 91 · 2–95 · 6). However, on adjusted analysis, the risk of FSGS recurrence in living related donor kidneys was significantly increased compared only with deceased donor kidneys (HR:2 · 2, 95% CI 1 · 34–3 · 64, *p* = 0 · 002) and not with living unrelated donor kidneys (HR:1 · 4, 95% CI 0 · 63–3 · 1, *p* = 0 · 4). There was no difference in the rates of recurrence free survival based on donor category among patients with MN (*p* = 0 · 2) or MCGN (*p* = 0 · 6) (Fig. 2).

### Increased glomerulonephritis recurrence in living related kidney transplants does not impair graft survival

**Fig. 3:**
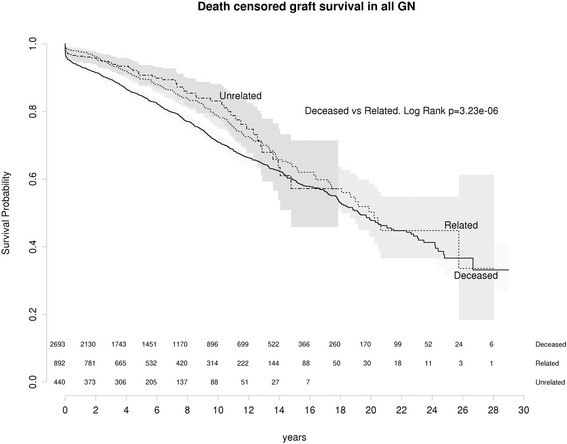
All GN: Death censored graft survival living related vs living unrelated vs deceased

We hypothesised that increased GN recurrence would have a negative effect on allograft survival and increased GN recurrence may mitigate the benefits of live donation. However, for all primary glomerulonephritides death censored graft survival (DCGS) was superior for live donor grafts compared to graft survival in deceased donor grafts (*P* < 0 · 001) and this survival advantage was maintained in living related donor grafts (Fig. 3).

**Fig. 4:**
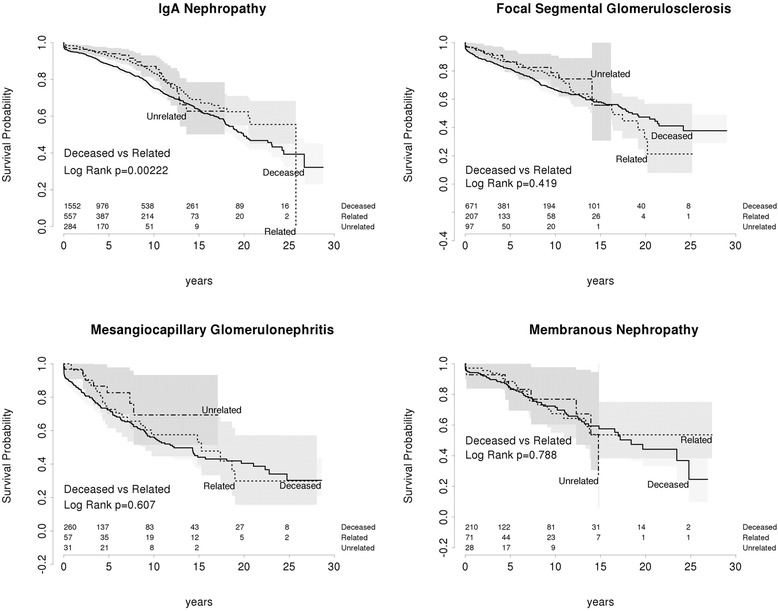
Death censored graft survival living related vs living unrelated vs deceased

In IGAN, living donor grafts had superior DCGS compared to deceased donor grafts (*p* = 0 · 002) (Fig. 4). In contrast, there was no significant difference in MN, FSGS or MCGN (Fig. 4). In FSGS at 5 and 10 years, DCGS showed a non-significant trend towards improvement for any live donor kidney (Fig. 4).

**Fig. 5:**
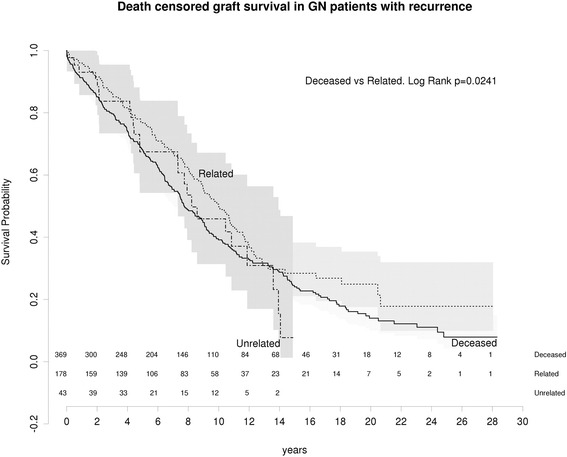
Death censored graft survival in grafts with recurrent disease living related vs unrelated from time of recurrence

We examined graft survival from time of recurrence (Fig. 5). There was no difference observed in graft survival of patients with recurrence of GN based on donor status. At 5 years, DCGS was 41 · 6% (95%CI 35 · 2–49 · 1) in deceased donor grafts and 52 · 9% (CI 44 · 8-62 · 4) in living related grafts. Therefore there is no evidence that living related grafts have more aggressive recurrent disease than deceased donor grafts.

### HLA mismatch does not associate with increased risk of glomerulonephritis recurrence

**Fig. 6:**
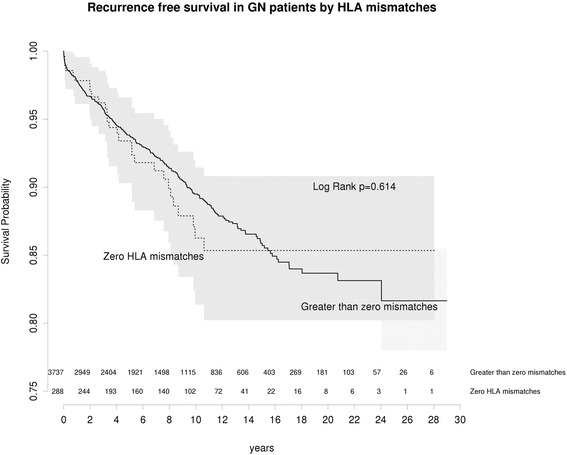
Recurrence free survival by HLA matching in living related or living unrelated grafts

We tested whether increased recurrence in IGAN and FSGS related to shared HLA antigens. The risk of recurrence was not significantly increased by in zero-mismatched grafts (Fig. 6). This suggests that the increased risk of recurrence associated with living related donation is not due to shared HLA but other heritable kidney-intrinsic factors.

## Discussion

Our study demonstrates an increased risk of primary GN recurrence within renal allografts from living related donors, mainly in patients with IGAN and FSGS. Increased risk with live donation has been reported in a number of studies of primary GN, and specifically in IGAN [16–21], FSGS [22–24] and MCGN [25–28]. This is the largest study of primary GN recurrence in transplantation on the effect of living related donation.

Current studies of the effect of donor status on recurrence and outcome are unclear. A meta-analysis of the effect of donor status reported a higher risk of IgA recurrence in living related grafts but a non-significant trend in the risk of graft loss [29]. The ERA-EDTA registry analysis [26] reported increased MCGN recurrence, but equivalent graft survival in living donor grafts, whether related or not. Other studies have likewise failed to find an association between live related donation and GN recurrence [30–33].

The mechanism of increased GN recurrence risk in living related grafts is unclear. This study implicates genetic risks for IGAN and FSGS manifesting in a kidney-intrinsic manner. However, it remains unclear if recurrence of IGAN is [34, 35] or is not [32, 36] more frequent in transplants with zero HLA mismatches. In this study we did not identify an elevated risk due to HLA, suggesting an alternative genetic mechanism. This study cannot exclude a role for minor HLA antigens.

Despite the elevated risk of GN recurrence demonstrated in grafts from living related donors, death-censored graft survival was still superior for patients with IGAN compared to deceased donors, and no difference was observed in death-censored graft survival in MN, FSGS or MCGN. Our study shows that, for those with a primary diagnosis of IGAN or FSGS, the benefit of live allograft donation is maintained regardless of the elevated risk of GN recurrence. This benefit does not appear to be present for patients with membranous or MCGN. Therefore, increased recurrence rates do not outweigh the superior outcomes associated with living donation, in agreement with previous studies [5, 7, 19, 26]. Based on our observations, live donor allografts remain the standard for transplantation in primary GN.

Whilst this is a large, well-characterised study population with 28 years follow-up, we acknowledge the limitations inherent in registry analysis. Despite these limitations, this study represents the most complete and well-populated registry analysis to date.

The increasing use of protocol biopsies post transplantation has the potential to alter the results of this analysis. The significance of histological evidence of recurrence in the absence of clinical evidence of glomerulonephritis is unclear. It may be argued that to adequately document the incidence and prevalence of post-transplant GN, a protocol biopsy regimen should be mandatory. Under those circumstances, it is highly likely that the GN recurrence rate would rise, but the overall impact on graft survival may fall. Whilst this might increase scientific rigour, it remains to be shown that such a policy can improve outcomes, both for recurrent GN and graft survival.

## Conclusions

In this Registry analysis of 7,236 patients with primary glomerulonephritis who received renal allografts between 1985 and 2013 we identified a significant increase in the risk of GN recurrence in patients suffering from IGAN and FSGS where donation occurs from a living related donor compared to a deceased donor. Despite this elevated risk of recurrent disease, the survival advantage of living related donation is maintained. We conclude that there is no reason to avoid living related donation in recipients with primary GN despite the elevated risks. Potential recipients should, however, be informed of the increased risk of disease recurrence when receiving an organ from a relative.

## Abbreviations

CI: Confidence interval
DCGS: Death censored graft survival
FSGS: Focal and segmental glomerulosclerosis
GN: Glomerulonephritis
HR: Hazard ratio
IGAN: IgA nephropathy
MCGN: Mesangiocapillary glomerulonephritis
MN: Membranous nephropathy
